# Micromirror Array with Adjustable Reflection Characteristics Based on Different Microstructures and Its Application

**DOI:** 10.3390/mi15040506

**Published:** 2024-04-08

**Authors:** Hao Cao, Zhishuang Xue, Hongfeng Deng, Shuo Chen, Deming Wang, Chengqun Gui

**Affiliations:** 1 The Institute of Technological Sciences, Wuhan University, Wuhan 430072, China; 2 Research Center of Graphic Communication, Printing and Packaging, Wuhan University, Wuhan 430072, China

**Keywords:** micromirror array, 3D lithography, reflection characteristics, adjustable

## Abstract

The conventional reflective optical surface with adjustable reflection characteristics requires a complex external power source. The complicated structure and preparation process of the power system leads to the limited modulation of the reflective properties and difficulty of use in large-scale applications. Inspired by the biological compound eye, different microstructures are utilized to modulate the optical performance. Convex aspheric micromirror arrays (MMAs) can increase the luminance gain while expanding the field of view, with a luminance gain wide angle > 90° and a field-of-view wide angle close to 180°, which has the reflective characteristics of a large gain wide angle and a large field-of-view wide angle. Concave aspheric micromirror arrays can increase the luminance gain by a relatively large amount of up to 2.66, which has the reflective characteristics of high gain. Industrial-level production and practical applications in the projection display segment were carried out. The results confirmed that convex MMAs are able to realize luminance gain over a wide spectrum and a wide range of angles, and concave MMAs are able to substantially enhance luminance gain, which may provide new opportunities in developing advanced reflective optical surfaces.

## 1. Introduction

Reflective optical surfaces have unique advantages such as a flexible system design and adjustable surface reflection characteristics [1,2,3,4,5], being extensively utilized in multiple applications such as in holographic displays, signal detection, beam shaping, communication systems, and microelectronics [6,7,8,9,10,11]. Hopkins JB et al. [4,12] fabricated a 1 mm square hexagonal planar micromirror array (MMA) and individually controlled each square hexagonal mirror unit with different mechanical designs to rapidly and stably generate angular tilts, thus controlling the position of the reflected light. Li Z et al. [13] obtained antireflective transparent surfaces consisting of silica nanocaps by the simple heat treatment of silica-coated monolayer colloidal crystal templates, which provided an effective reduction in the reflectivity of the reflected light. Inspired by the moth eye, Yue gang Fu and team [14,15,16,17] prepared microstructural reflective surfaces of cylinder, cone, and circular hole shapes by using reactive ion etching to regulate the reflectivity of such surfaces through microstructures for anti-reflection effects, or through other bionic micro–nano structures to modulate the reflected light properties.

However, multiple studies focused on the increase/reduction of reflectivity for a certain direction and the realization of additional driving forces [18,19,20]. Currently, no desirable solution is available to regulate the reflection characteristics of reflective optical surfaces, such as working ranges, luminance gain intervals, and gain values, by using microstructures. And existing means of preparing reflective optical surfaces, such as precision machining, reactive ion etching, the machining degree of freedom, precision, efficiency, and costs of these microstructure preparation methods cannot be satisfied simultaneously, and the processable format is small each time, making industrial applications difficult [21,22,23].

Herein, different microstructures prepared by means of 3D lithography are used to modulate reflective properties. We successfully obtained reflective optical surfaces with diverse characteristics without the need for complex drive systems, and they have excellent color performance and image quality. Convex MMAs have a large gain wide angle and field of view wide angle, while concave MMAs have a high gain peak. Finally, we attempted industrial-level production and tested the actual application in the projection display segment, and results showed that we obtained products with excellent performance with lower production costs.

## 2. Design and Preparation of MMA

Light can “disappear” out of the void after it enters moth eyes. Studies indicate that the surface hexagonal convex microstructure produces absorptive effects on the incident light. Inspired by this, an aspherical micromirror, a microstructure, was prepared as a reflected surface, consequently creating a large-format MMA. The caliber of the aspherical micromirror is 80 μm and its height is 10 μm; the surface aluminum layer thickness is approximately 60 nm. After incident light is reflected by the MMA, the reflected light is regulated by the reflected surface for diverse effects, such as large signal gain, wide coverage, large color gamut, and small chromatic differences.

**Figure 1:**
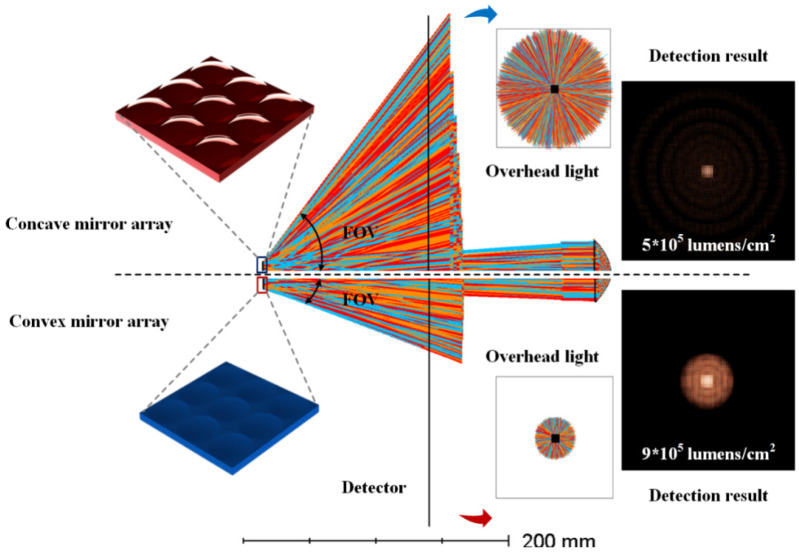
Optical simulation model and results.

Figure 1 shows the optical simulation model and results for different MMAs. The simulation model is composed of a light source, a collimating plane, an MMA, and a detector. The object distance between the light source and the micromirror is 0.2 m, while the interval between the detector and the micromirror is 0.1 m. The simulation result is as shown in the right side of Figure 1. It can be observed that the collimating beam can maintain high illuminance, at 9 × 10^5^ lm/cm^2^ and 5 × 10^5^ lm/cm^2^, respectively, after the beam is regulated by two MMAs, while the illuminance of an ordinary planar mirror can only reach 2 × 10^5^ lm/cm^2^ under the same conditions. In addition, the concave micromirror is superior to the convex micromirror in terms of optical gain, while the convex micromirror is superior to the concave micromirror in terms of the visual angle width as it can reach a luminance gain wide angle above 100°. The optical simulation results verify that the microstructural array is able to modulate the characteristics of the reflective optical system, in which the concave micromirror is able to significantly enhance the luminance gain and the convex micromirror is able to expand the gain range (the process of simulation is shown in the Supporting Information).

**Figure 2:**
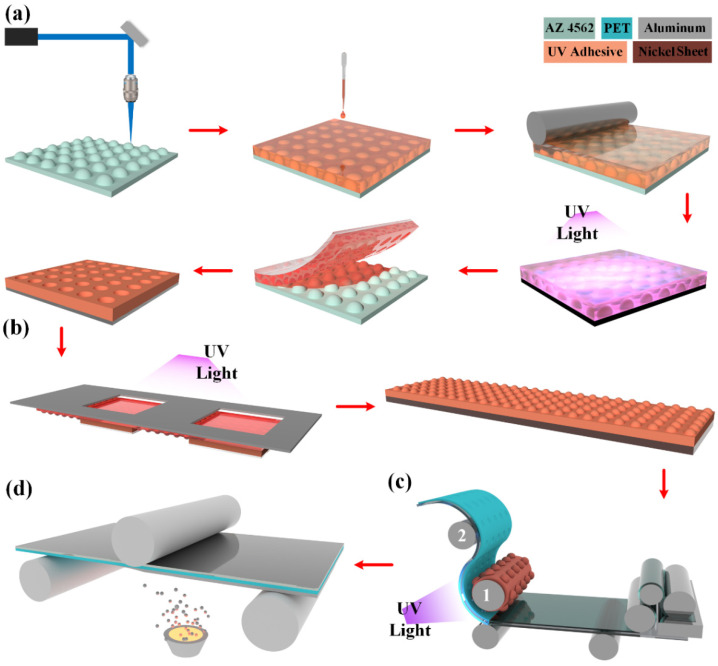
Manufacturing process of MMA: (**a**) 3D lithography; (**b**) preparation of templates; (**c**) UV transfer; (**d**) aluminized film.

The entire MMA preparation process was mainly composed of 3D lithography for the microstructure, UV transfer, and plating a reflective coating. As shown in Figure 2, the laser beam was focused on the photoresist surface for exposure; the photoresist of various depths was exposed by adjusting the exposure dose of the laser beam, and the microstructures of various heights were obtained after development. During the exposure, the laser beam passed through the integrated optical system. In the system, the attenuator and the diaphragm regulated the light intensity and numerical aperture; the regulated laser beam was focused on the photoresist via the collecting mirror as a light source to complete the exposure of the photoresist. Gray-level photoresist is sensitive to exposure power; diverse exposure powers result in different exposure depths of the photoresist. Therefore, different photoresist depths can be exposed by regulating the exposure power to obtain a 3D structure with an ideal shape. Electrical modulation was adopted for the device used in this study to regulate the exposure power; if the gray-level exposure of a light beam reaches 4096, the result is that 4096 microstructures with diverse heights and gradients can be obtained.

**Figure 3:**
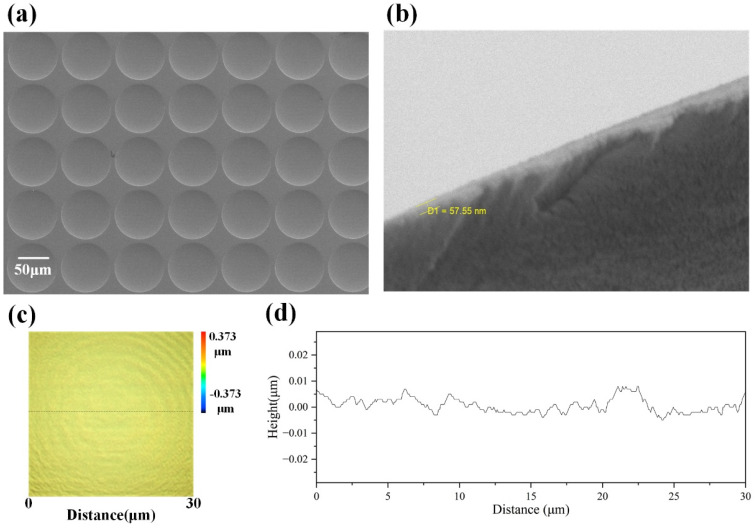
Characterization result of MMA: (**a**,**b**) are the SEM images of the MMAs and the surface aluminum layers; (**c**,**d**) are the height change in the central area.

After 3D lithography, UV transfer was performed to obtain a template. First of all, UV adhesive was dropped onto the prepared microstructural slab; the UV adhesive was solidified through ultraviolet light polymerization, and a UV slab with microstructures was obtained after demolding; afterwards, UV exposure was performed for different locations to make large-format UV slabs. Through large-format lithography, a 100-inch MMA could be made by two splicing, not only to improve the production efficiency, but also to reduce the structural losses during splicing. A roll-to-roll approach was employed for the UV compression molding transfer: the template was placed on Cylinder 1 and the PET/PMMA flexible film was placed on Cylinder 2. When the PET flexible film rotated, the UV adhesive was applied to the film; microstructures took shape after the film passed the template, and ultraviolet light polymerization was performed. Finally, thermal evaporation was employed for plating the aluminum film as a reflecting layer. Figure 3a,b show the MMAs and the SEM image of the surface aluminum layers. Figure 3c,d demonstrate the height variation in the center region (30 μm × 30 μm) with a surface roughness of 8.5 nm, which meets the requirements for the use of optical devices.

## 3. Results and Discussion

### 3.1. Analysis of Reflection Characteristics

**Figure 4:**
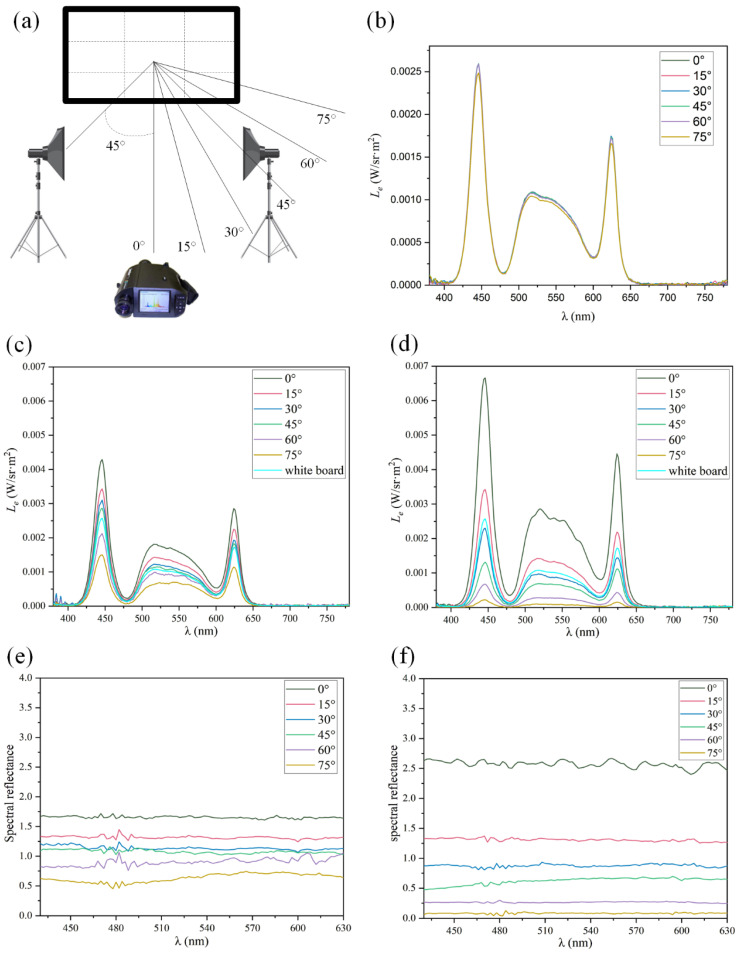
(**a**) is the schematic diagram of the testing conditions; (**b**) shows the reflected light radiance of the standard reflective white board at various angles; (**c**) demonstrates the reflected light radiance of the convex MMA at different angles; (**d**) shows the reflected light radiance of the concave MMA at diverse angles; (**e**) demonstrates the spectral reflectivity of the convex MMA at various angles; and (**f**) is the spectral reflectivity of the concave MMA at different angles.

As shown in Figure 4a, the light source D65 was adopted to illuminate the screen from left and right sides of the screen from an angle of 45°, and a PR-705 spectrophotometer (hereinafter referred to as “PR-705”) was utilized to collect the reflected light radiance at 0°, 15°, 30°, 45°, 60°, and 75° respectively. D65 and PR-705 were both positioned 2 m from the MMA. The D65 intensity was adjusted to third gear. PR-705 receives reflectance spectra in the range of 380–780 nm. The results are shown in Figure 4b–f.

Figure 4b shows the results of a standard white board. It can be observed that the white board has nearly constant reflected light: both curves have three maximum values and two minimum values; the three maximum values appear at (446 nm, 0.0026 W/sr·m^2^), (518 nm, 0.0011 W/sr·m^2^), and (624 nm, 0.0017 W/sr·m^2^), respectively, while the two minimum values appear at (480 nm, 0.00014 W/sr·m^2^) and (600 nm, 0.00032 W/sr·m^2^). The curve shapes are the same as the spectral distribution of light source D65 because the standard white board is a typical diffuse reflection surface and the reflectivity is approximate to 1 in all directions to reflect the incident light from the light source to all directions in a lossless manner. The experimental results indicate the Lambertian characteristics of standard reflective white boards.

Figure 4c,e demonstrate the results for the convex MMA. It can be found that, within the scope of 380–780 nm visible light, the array has the highest radiance at 0°; the curve shape is consistent with the light source spectrum, being smooth and reaching the maximum value of 0.0043 W/sr·m^2^ at 446 nm. The array starts declining at 15° and the curve shape is consistent with the light source spectrum, being smooth; at 30°, it continues to decline with a slow amplitude, and the curve shape is consistent with the light source spectrum, being smooth. It declines slightly at 45°, and the reflected light radiance at each waveband is slightly higher than that of the standard white board. The curve shape is consistent with the light source spectrum, but there is a small fluctuation in the middle waveband; it continues to decline at 60°, and the reflected light radiance at each waveband is slightly lower than that of the standard white board. It has the lowest radiance at 75°, reaching the minimum value of 0.000041 W/sr·m^2^ at 480 nm. This is because, at 0°, the convex aspheric micromirror reflects incident light to a large range, but the structureless area has the strongest reflection; therefore, the radiance is highest at 0°, and the overall radiance has large gains compared with those of the white board. After it deflects to 15°, the convex aspheric micromirror can reflect incident light into the scope. The reflected light is relatively strong, while the reflectivity of the structureless area declines and the radiance is lower, but it still has gains. After it deflects to 30° and 45°, the convex aspheric micromirror can reflect incident light into the scope and the reflected light is strong, so the radiance declines in turn, but it still has gains. After it deflects to 60° and 75°, the convex aspheric micromirror can still reflect incident light into the scope, but the reflected light is not as strong as before and the structureless area can barely reflect, so the radiance is lower than that of the white board, but it can still obtain easily identifiable reflected light. The experimental results indicate that the gain wide angle of the convex MMA is >90° and its working interval is approximate to 180°.

Figure 4d,f show the results for the concave MMA. It can be observed that, within the scope of 380–780 nm visible light, the array has the highest radiance at 0°; the curve shape is consistent with the light source spectrum, reaching the maximum value of 0.0067 W/sr·m^2^ at 446 nm. The array starts declining at 15°, and the curve shape is consistent with the light source spectrum, being smooth. At 30°, it continues to decline with a slow amplitude; the reflected light radiance of the wavebands has small differences with that of the standard white board at this moment, and the curve shape is consistent with the light source spectrum. It declines at 45° and continues declining at 60°, reaching the minimum value of 0.000039 W/sr·m^2^ at 480 nm. It declines to the lowest level at 75°; the curve shape is nearly a straight line and the values of all of the wavebands are approximate to 0. The reason is that, at 0°, the concave aspheric micromirror has a convergence function whereby it converges incident light to the signal receiver and the structureless area is a mirror reflection, and it has the highest radiance because of the strongest reflection at that moment. The overall radiance has large gains compared with those of the white board, but the spectrum curve has some fluctuations; after it deflects to 15°, the reflected light of the concave MMA can still reach the scope, and the light is strong with a large amplitude of declination, so the radiance is lower, but it still has some gains. After it deflects to 30°, the reflected light converged by the concave MMA can still reach the scope, but the light declines, so the radiance declines to less than the white board. After it deflects to 45° and 60°, the array acts as a shelter; very little reflected light from the array can reach the scope, so the radiance is reduced dramatically and is smaller than that of the white board. After it deflects to 75°, the shelter function of the array is more apparent, and the radiance is nearly 0. The experimental results indicate that compared with the convex MMA, the concave MMA has a smaller gain wide angle and working interval; however, its maximum gain value can reach 2.66. This confirms that reflective optical surfaces formed by different microstructures have different reflective properties.

### 3.2. Analysis of Color Gamut and Chromatic Differences

**Figure 5:**
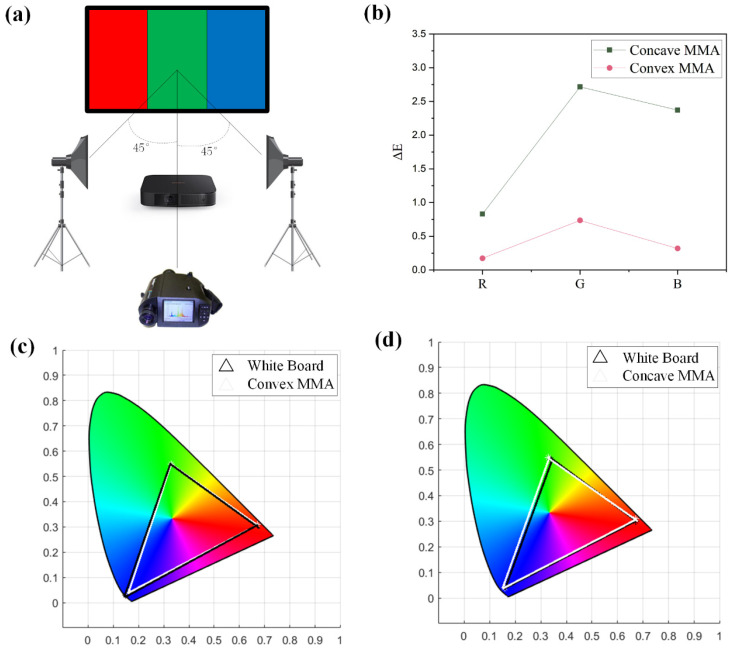
(**a**) is the schematic diagram of testing conditions; (**b**) shows the chromatic differences of the convex/concave MMAs; (**c**) demonstrates the color gamut of the convex MMA; and (**d**) is the color gamut of the concave MMA.

A Photo Research 705 spectrum radiometer was utilized for the screen-reflected color measurement of the MMAs. A non-contact color measurement was conducted. The 45°/0° color measurement geometrical conditions, recommended by CIE, were adopted to guarantee the illumination uniformity of the screen surface. Two standard D65 light sources were placed at both sides of the measured screen, each forming a 45° angle with the normal of the screen; PR705 was perpendicular to the screen, and the measurement conditions are shown in Figure 5a. D65 and PR-705 were both positioned 2 m from the MMA. The D65 intensity was adjusted to third gear. Light of 700 nm, 546.1 nm, and 435.8 nm wavelengths were projected onto the MMA. A standard white board was first measured to obtain the spectral radiance values. Under the same conditions, the colored light reflected by the MMA was measured.

Tristimulus values were employed to calculate the chromatographic coordinates of the colors so as to calculate the color gamut range covered by the colors reflected by the screen (the process of the calculation is shown in the Supporting Information). Figure 5c,d show the color gamut ranges of the convex/concave MMAs, respectively. The white triangular curve is the color gamut range of the standard white board, while the black triangle is that of the MMA. The figures show that the chromaticity coordinates of the colors reflected by the convex MMA are (0.67, 0.31), (0.33, 0.55), and (0.15, 0.03), respectively; the chromaticity coordinates of the colors reflected by the concave MMA are (0.67, 0.30), (0.34, 0.54), and (0.16, 0.04), respectively; and the chromaticity coordinates of the standard white board are (0.67, 0.31), (0.33, 0.55), and (0.16, 0.04), respectively. It can be found that the color gamut range of the convex MMA is nearly the same as that of the white board, while the concave MMA is different in the green and red parts. Generally, their color gamut ranges are consistent, and the color gamut covers 99% of the sRGB. The reason is that the color reproduction is not compromised by the MMA preparation system. This proves that microstructures do not reduce color gamut ranges when modulating the luminance gain, gain ranges, and working ranges.

A chromatic difference calculation was made to verify the color reproduction capacity of the MMAs (the process of the calculation is shown in the Supporting Information), and the results are shown in Figure 5b. The chromatic differences of the convex MMA in the red, green, and blue wavebands are 0.1741, 0.7356, and 0.3196, respectively; the chromatic differences of the concave MMA in the red, green, and blue wavebands are 0.8313, 2.7163, and 2.3720, respectively. The chromatic differences of the convex MMA are less than 0.75 and are extremely small, indicating that the MMA has a strong color reproduction capacity. The chromatic differences of the concave MMA are less than 2.72; the chromatic differences in the green and blue wavebands are larger, but are within acceptable ranges. Generally speaking, the MMAs have different reflective characteristics and, at the same time, acceptable small chromatic differences in reflected colors and a desirable color reproduction capacity.

### 3.3. Analysis of Image Quality

Besides the luminance and color properties, reflected image quality is an important evaluation parameter for reflective optical systems as well. Human visual perceptions were combined with four image quality evaluation indicators, i.e., edge intensity (EI), average gradient (AG), information entropy (EN), and differential mean opinion score (DMOS), to evaluate the reflective images of MMAs and the Fresnel reflection screen (the meaning of the four quantitative indicators is provided in the Supporting Information).

**Figure 6:**
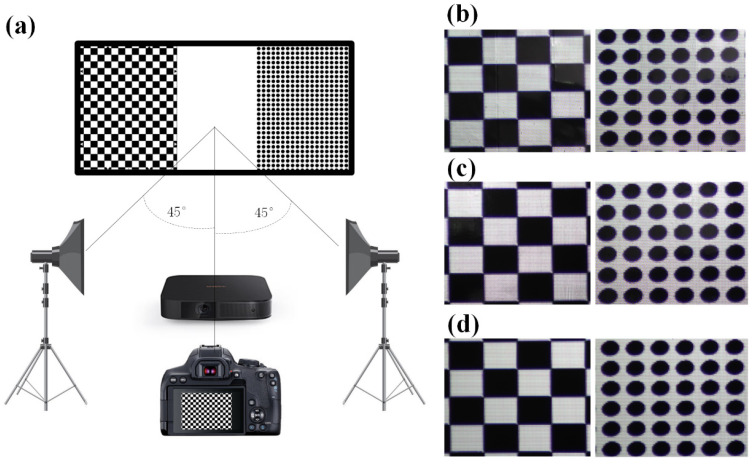
(**a**) is the schematic diagram of testing conditions; (**b**) is about the test results of the convex MMA; (**c**) shows the test results of the concave MMA; and (**d**) demonstrates the test results of the Fresnel optical screen.

As shown in Figure 6a, the projected images were changed to a standard square chessboard and a round array test to analyze the imaging properties of diverse geometric structures. A digital camera (Nikon D3200, Nikon, Tokyo, Japan) was used to take photos. The D65 light source and the digital camera were both positioned 2 m from the MMA. The D65 intensity was adjusted to third gear. Figure 6b,c show the results. It is observed that the square and round images have clear and explicit edges, and the definitions and contrasts of the edges are high. Both convex and concave MMAs do not result in distortion as a result of image off-centering. No geometric distortions were found in the convex and concave MMAs, while the imaging effects were consistent with the Fresnel screen imaging effects produced by industrial production. The reason for this is that the processing system in the paper can perfectly control structural shapes and meet usage requirements. As mentioned above, the length and width of each pixel in a large-format MMA are 80 μm and the resolution can reach 317.5/inch. Therefore, they can meet the requirements of existing practical applications.

An objective evaluation method was employed for quantitative analysis to further evaluate the image quality. Four image quality indicators were employed to evaluate the image quality. The evaluation results are shown in Table 1.

Square image test: It can be observed that the EI value of the convex MMA is equivalent to that of Fresnel (the width of the unit structure is 120 μm and the height is 10 μm), while that of the concave MMA is slightly larger than that of Fresnel, indicating that MMAs composed of microstructures meet or even slightly exceed Fresnel levels in EI. The AG value of the convex MMA is equivalent to that of Fresnel, while that of the concave MMA is slightly less than that of Fresnel, indicating that MMAs composed of microstructures meet Fresnel levels in AG. The EN value of the convex MMA is the largest, followed by the concave MMA, and that of the Fresnel is the smallest. It can be observed that the DMOS value of Fresnel is the largest, followed by the concave MMA, and that of the convex MMA is the smallest, indicating that the Fresnel-reflected image has the lowest quality and that of the convex MMA has the highest. Round image test: It can be observed that the convex MMA has the highest EI value, the concave MMA has the lowest EI value, and Fresnel is between the two. This situation applies to AG values; however, the concave MMA has the largest EN value, followed by that of the convex MMA. The differences between them are quite small, and the EN value of Fresnel is the lowest. Fresnel DMOS is the largest, followed by the concave MMA, and the convex MMA has the smallest DMOS. Generally, all of the indicators of the convex MMA in the round array test are satisfactory, followed by the concave MMA, while those of Fresnel are the worst. The reason for this may be that the Fresnel structure blocks light to a certain extent and the working range is small, resulting in the worst overall image quality. The luminance gains of the convex MMA are within an interval with smooth changes; the working range is large and the color reproduction capacity is satisfactory, making the overall image quality high. The concave MMA has good color reproduction capacity and the luminance gains are large, but the gain changes are steeper and the working range is smaller than that of the convex MMA. Therefore, its overall image quality is ranked second place. According to the subjective and objective image quality evaluations, the designed concave and convex MMAs have a better imaging effect.

## 4. Industrial-Level Production and Applications

**Figure 7:**
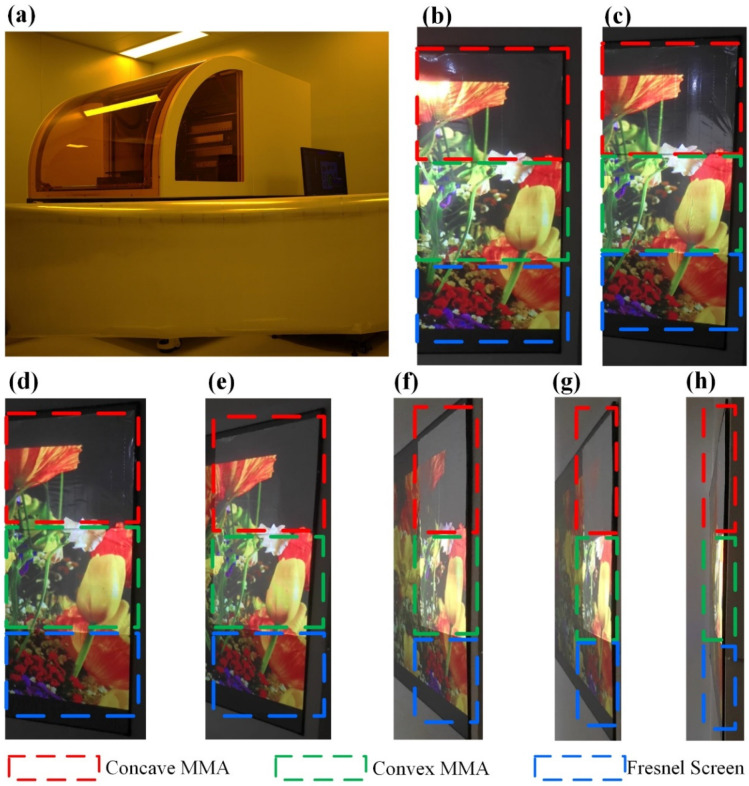
(**a**) is the large-scale MMA prepared for the paper; (**b**–**h**) display the image photographed at 0°, 15°, 30°, 45°, 60°, 75°, and ~90°, respectively.

In this study, an attempt was made to produce the designed convex/concave MMAs at an industrial-level scale, as shown in Figure 7a, and were applied to the projection display segment for comparison with a commercially priced Fresnel optical projection screen. The images were projected to the three screens and photographed at 0°, 15°, 30°, 45°, 60°, 75°, and ~90°, respectively. The results are shown in Figure 7b–h.

It can be observed that, at 0°, the concave MMA has the highest image luminance, the convex MMA image luminance is ranked second place, the colors are uniform and exquisite, and the overall effects are desirable. The Fresnel optical screen has the darkest image that is slightly dim; at 15°, the concave MMA has the highest image luminance, but the luminance is lower than that at 0°. The convex MMA image luminance is ranked second place, the colors are uniform and exquisite, and the overall effects are good. The Fresnel optical screen has the darkest image that is slightly dim. At 30°, the concave MMA image luminance continues to decline and the convex MMA layer image luminance is lower as well, but the overall effects are good. The Fresnel optical screen has the darkest image with distortion at some dim parts; at 45°, the concave MMA image luminance continues to decline and becomes dim. At this moment, the convex MMA image has the highest luminance, with overall good image effects. The Fresnel optical screen has the darkest image, with larger image distortion; at 60°, the concave MMA has the darkest image luminance, with poor imaging effects. The convex MMA image remains bright, with good overall effects. The Fresnel optical screen image is the darkest, with larger distortion; at 75°, the concave MMA image luminance continues to decline, and it is difficult to identify the image with poor imaging effects. The convex MMA image has the highest luminance, making it the only bright screen with good overall effects. The Fresnel optical screen image is dark, with larger distortion; at 90°, it is difficult to identify the concave MMA and the Fresnel optical screen, while the convex MMA image has the highest luminance, making it the only sample with signals. Overall, the convex MMA has the best performance when applied to projection displays; concave MMA can be used for retro-reflective marking, where the reflected light is brighter and can be recognized at longer distances.

Table 2 shows a detailed comparison of the MMA with the Fresnel optical screen. Three-dimensional lithography was employed for the convex MMA optical screen, and the machining accuracy and surface roughness were found to be superior to precision machining for existing Fresnel optical screens [24,25]. The machining speed for the convex MMA screen is 233% of that for the latter; moreover, the machining process is simple and the machining freedom is high, as 4096 gradients are available on Axis Z. Existing equipment can form an 800 mm × 800 mm microstructure surface at a time; preparing large-format products has always been a constraint for precision machining. The machining difficulty increases with the increase in the format, while 3D lithography can easily solve the problem of preparing large-format products, not only improving the machining, and R&D efficiency, but also lowering the costs. This shows that the technology system of “3D lithography + UV compression molding transfer” can substantially lower production costs and improve R&D, being of great importance to scientific research and engineering manufacturing.

## 5. Conclusions

In summary, the microstructures prepared using 3D lithography are utilized to modulate reflective properties without additional power sources. The luminance gain wide angle of the convex MMA is >90° and the visual angle width is approximate to 180°; with chromatic differences < 0.75 and small geometric distortions, the MMA has reflection characteristics such as wide-range gains and large-interval working ranges. The maximum surface gain of the concave MMA can reach 2.66; with a chromatic difference < 2.72 and small geometric distortions, the MMA has high gain reflection characteristics. This has been verified in the field of laser projection and better results were achieved: we attempted industrial-level production and applied the microstructures to projection display segments with better performance and lower processing costs compared to existing mainstream optical screens. The research in this paper may provide different ideas for the development of innovative reflective optical systems and the promotion of efficient R&D and actual production, providing a highly valuable solution for industrial applications.

## 6. Experimental Section

Fabrication: We uploaded the design drawings of the MMA into the 3D lithography equipment (Pico Master 100, 4PICO, Sint-Oedenrode, The Netherlands), and prepared a 1 mm thick glass coated with the 12 μm photoresist (AZ4562, Merck, Darmstadt, Germany). The glue application speed was 650 r/min. Next, the glass coated with photoresist was relaxed for 30 min to release the air bubbles in the photoresist. We baked the photoresist at 90 °C for 50 min using a slow heating process. After 10 min cooling, we put the glass coated with the photoresist into the 3D lithography equipment. In the lithography process, the lithography spot was 550 nm, the step was 275 nm, the speed was 100 mm/s, and the exposure intensity was 800 mj/cm^2^. Finally, the photoresist was developed for 6 min after the laser exposure. The developer used was AZ400K (AZ400K, Darmstadt, Germany). UV adhesive (Kangdexin Composite Material Group, MAC91A, Beijing, China) was applied to the stencil and irradiated for 35 s using a UV lamp (Kangdexin Composite Material Group, S901, Beijing, China). Roll-to-roll embossing was carried out with self-developed equipment: the mold temperature was 65 °C, the glue filling rate was 0.5 m/s, and the curing time was 35 s.

Characterization: SEM images of the gold-sputtered samples were taken by a field-emission scanning electron microscope (MIRA3, Tescan, Brno, Czech Republic). Surface roughness data were obtained by confocal microscopy (VK-X1000, Keyence, Osaka, Japan). A spectrophotometric radiation brightness meter was also utilized (PR-705, Photo Research, Syracuse, NY, USA).

## Figures and Tables

**Table 1:** Results of quantitative analysis.

	Square Image Test Results				Round Image Test Results			
Microstructure	EI	AG	EN	DMOS	EI	AG	EN	DMOS
Convex	61.42	5.51	6.28	39.50	89.61	8.08	6.95	27.28
Concave	61.56	5.28	6.26	43.39	80.95	7.31	6.95	27.89
Fresnel	61.42	5.50	6.12	46.74	84.04	7.56	6.74	30.06

**Table 2:** Comparison of various optical screens and machining methods.

Comparative Item	MMA Optical Screen	Fresnel Optical Screen
machining method	3D lithography	precision machining
accuracy	0.3 μm	1.0 μm
surface roughness	8.5 nm	50 nm
machining speed	8929 mm^2^/h	3828 mm^2^/h
maximum format	800 × 800 mm	determined by the mold
machining freedom	designed at will on Axis Z	determined by the mold
working range	~180°	>30°
gain range	>90°	~30°

## Data Availability

The original contributions presented in the study are included in the article/supplementary material, further inquiries can be directed to the corresponding authors.

## Supplementary Materials

The following supporting information can be downloaded at: https://www.mdpi.com/article/10.3390/mi15040506/s1, Section S1. The process of Zemax simulation; Section S2. Determine the dimensional parameters of the MMA; Section S3. Calculation method of color gamut; Section S4. Calculation of chromatic differences; Section S5. The meaning of the four quantitative indicators. Figure S1: Simulation results of MMA simulation at different depths: (a–f) are simulation results at depths of 1 μm, 2 μm, 4 μm, 6 μm, 8 μm, and 10 μm, respectively. Figure S2: The change rule of gain with caliber and depth.
