# Supplementary material for: Micromirror Array with Adjustable Reflection Characteristics Based on Different Microstructures and Its Application
Source: Micromachines (Basel). 2024 Apr 8;15(4):506. doi: 10.3390/mi15040506 (PMC11052270; doi:10.3390/mi15040506)
Supplement: Supplementary file 1 [file micromachines-15-00506-s001.zip › micromachines-2936911-supplementary.pdf]

Supporting Information

Micromirror array with adjustable reflection characteristics based on different microstructures and its application

Hao Cao[1], Zhishuang Xue[2], Hongfeng Deng[1], Shuo Chen[1], Deming Wang[1], Chengqun Gui[1] *

1.The Institute of Technological Sciences, Wuhan University, Wuhan 430072, China
2.Research Center of Graphic Communication, Printing and Packaging, Wuhan University, Wuhan 430072, China
Correspondence: Hao Cao (2021106520024@whu.edu.cn) or Chengqun Gui (cheng.gui@whu.edu.cn)

S1. The process of Zemax simulation

The main simulation steps are as follows:

(1) Using SolidWorks to establish an MMA model and import it into Zemax; the dimensional proportion of the MMA to the actually prepared array is 1: 1;

(2) Configure the light source, detector, and collimating plane; the light source is IESNA standard point light source, the luminous intensity is 10^6lm, the number of light rays is 2×10^8, the detector's pixel is 20 um×20 um, the size is 200 mm ×200 mm, and the collimating plane is a concave mirror with aspheric degree of -0.2;

(3) During the simulation, the light from the point light source enters the two MMAs and goes back to the detector after passing the collimating plane;

(4) Calculating the visual angle width and optical gains. The MMA visual angle width is calculated as per the light-emitting area proportion of X, Y interfaces and the geometrical relationship between the micro mirror and detector on the X, Z interfaces; illumination is calculated by importing the detector's light ray data into MATLAB and such data are compared with ordinary planar reflection mirrors to calculate optical gains.

S2. Determine the dimensional parameters of the MMA

In order to determine the specific structural characteristic parameters of the micro mirror, the simulation analysis of micro mirror arrays with different apertures and depths is carried out here. The concave micro mirror arrays are selected as the objects, whose aperture variations are 60 μm-80 μm and depth variations are 2 μm-10 μm, respectively. The reason for selecting this range is to consider the process window and visual effect of the later large-area transfer, the depth is too high, in the process of splicing templates and large-scale transfer will lead to too much structural loss, and cannot achieve the designed effect; the aperture is too large, greater than the human eye resolution of 100μm will lead to poor visual effect.

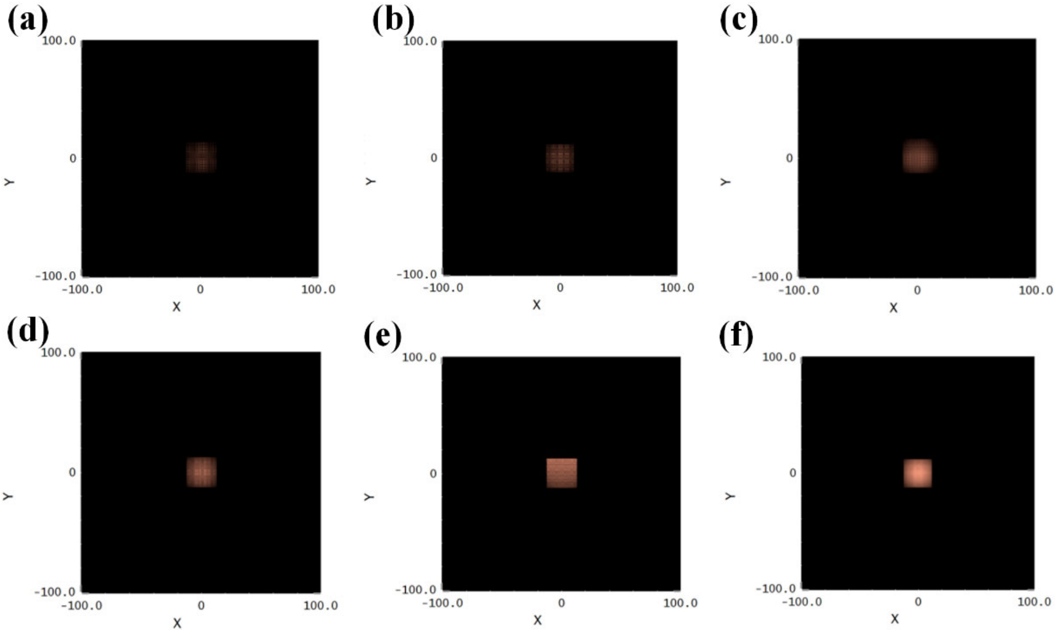

Figure S1. Simulation results of MMA simulation at different depths: (a)-(f) are simulation results at depths of 1 μm, 2 μm, 4 μm, 6 μm, 8 μm, and 10 μm, respectively

The simulation model and analysis method are similar to the previous one, as shown in Figure S1 for the peak illuminance results of the micro mirror arrays of 80 μm aperture with depths of 2 μm, 4 μm, ..., and 10 μm, respectively, and it can be found that with the same aperture of the micro mirror arrays, the peak illuminance rises and the gain will be increased with the increase of the depth.

Similarly, the micro mirror arrays with apertures of 70 μm and 60 μm apertures at different depths were analyzed, and the results are shown in Figure S2, where the gain increases with increasing aperture and depth. Therefore, in this paper, as far as the process and visual effect allow, the aperture of the micro-reflector is selected to be 80 μm and 10 μm.

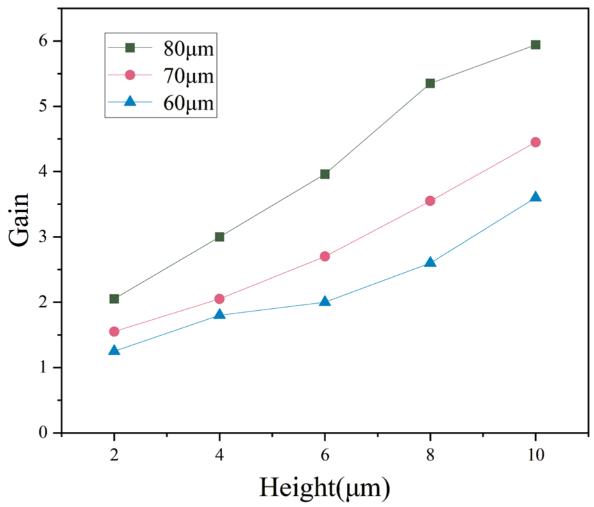

Figure S2. The change rule of gain with caliber and depth

S3.Calculation method of color gamut

In the paper, Formula (1) was utilized to process the data collected by PR-705 to obtain the spectral reflectivity of the screen surface.

$$R_t = R_w \times \frac{SPD_t}{SPD_w}$$

Where, Rt and Rw represent the spectral reflectivity of the screen surface and that of the standard white board respectively; SPDt and SPDw represent their corresponding spectral irradiances respectively. The tristimulus values X, Y, and Z of the measured screen were obtained through calculation:

$$\begin{cases} X = \int_{380}^{780} S(\lambda)R_t(\lambda)\bar{x}(\lambda)d\lambda \\ Y = \int_{380}^{780} S(\lambda)R_t(\lambda)\bar{y}(\lambda)d\lambda \\ Z = \int_{380}^{780} S(\lambda)R_t(\lambda)\bar{z}(\lambda)d\lambda \end{cases}$$

Where, $S(\lambda)$ represents the corresponding spectral power distribution of the light source, and $\bar{x}(\lambda)$ 、 $\bar{y}(\lambda)$ and $\bar{z}(\lambda)$ are the tristimulus values of CIE1931 standard observer spectrum. For the calculation of color gamut, the tristimulus values were utilized to calculate the chromatographic coordinates of the colors to calculate the color gamut coverage of the colors reflected by the screen.

S4.Calculation of chromatic differences

A calculation of chromatic differences was conducted to verify the color reproduction capacity of the MMAs. By using the CIE 1931-XYZ standard chroma system, the colors measured by the array surface were converted to the CIELAB, and the conversion process is as follows:

$$L^* = 116 f(Y/Y_n) - 16$$
$$a^* = 500[f(X/X_n) - f(Y/Y_n)]$$
$$b^* = 200[f(Y/Y_n) - f(Z/Z_n)]$$

Where, X_n, Y_n, and Z_n are the tristimulus values of CIE standard light source illuminated on the full diffuse reflector and to the eyes of the observer, and the $f(\cdot)$ expression is:

$$f\left(\frac{X}{X_n}\right) = \begin{cases} \left(\frac{X}{X_n}\right)^{1/3} & \left(\frac{X}{X_n}\right) > (\frac{24}{116})^3 \\ \left(\frac{841}{108}\right)\left(\frac{X}{X_n}\right) + \frac{16}{116} & \left(\frac{X}{X_n}\right) \le (\frac{24}{116})^3 \end{cases}$$

$$f\left(\frac{Y}{Y_n}\right) = \begin{cases} \left(\frac{Y}{Y_n}\right)^{1/3} & \left(\frac{Y}{Y_n}\right) > (\frac{24}{116})^3 \\ \left(\frac{841}{108}\right)\left(\frac{Y}{Y_n}\right) + \frac{16}{116} & \left(\frac{Y}{Y_n}\right) \le (\frac{24}{116})^3 \end{cases}$$

$$f\left(\frac{Z}{Z_n}\right) = \begin{cases} \left(\frac{Z}{Z_n}\right)^{1/3} & \left(\frac{Z}{Z_n}\right) > (\frac{24}{116})^3 \\ \left(\frac{841}{108}\right)\left(\frac{Z}{Z_n}\right) + \frac{16}{116} & \left(\frac{Z}{Z_n}\right) \le (\frac{24}{116})^3 \end{cases}$$

L*a*b* was used as the measured value of the MMA. The chromatic difference $CIE\ \Delta E^*_{1976}$ between the measured values corresponding to the MMA and the standard value of the standard white board was calculated respectively, and the expression is:

$$\Delta E^*_{1976} = \sqrt{(\Delta L)^2 + (\Delta a)^2 + (\Delta b)^2}$$
$$\Delta L = L_s - L,\ \Delta a = a_s - a,\ \Delta b = b_s - b,$$

Where, Ls, as, and bs represent the L*a*b* values obtained by measuring the standard white board, and L, a, and b represent the actual measured values corresponding to the MMA respectively.

S5.The meaning of the four quantitative indicators

Edge intensity (EI) was employed to describe the edge contour information of an image. The gradient directions in the pixels were calculated, and the differential stimulus values in the vertical and horizontal directions were calculated. A high-quality image has sharper edges and larger gradients; Average gradient (AG) refers to the differences of changes of the gray levels in image boundary or at the both sides of hatching line, namely the rate of change for gray level, reflecting the tiny detail differences and texture change characteristics in images, and edge definition and contrast are evaluated to measure image definitions; Information entropy (EN) refers to the quantity of average information content contained in images; the larger the entropy is, the more information the image will contain; Differential mean opinion score (DMOS) refers to the mean value of subjective score differences for the original image and the image reflected by the screen; the larger the DMOS is, the larger the score differences is, and the lower the quality of the image reflected by the screen is.
